# Older Adult Perspectives on Physical Activity and Exercise: Voices From Multiple Cultures

**Published:** 2004-09-15

**Authors:** Basia Belza, Julie Walwick, Sheryl Schwartz, James LoGerfo, Sharyne Shiu-Thornton, Mary Taylor

**Affiliations:** Department of Biobehavioral Nursing and Health Systems; Health Promotion Research Center, School of Public Health and Community Medicine, University of Washington, Seattle, Wash; Health Promotion Research Center, School of Public Health and Community Medicine, University of Washington, Seattle, Wash; Health Promotion Research Center, School of Public Health and Community Medicine, University of Washington, Seattle, Wash; Department of Health Services, School of Public Health and Community Medicine, University of Washington, Seattle, Wash; School of Nursing, University of Washington, Seattle, Wash

## Abstract

**Introduction:**

Increasing physical activity is a goal of *Healthy People 2010*. Although the health benefits of physical activity are documented, older adults are less physically active than any other age group. The purpose of this study was to examine barriers and facilitators to physical activity and exercise among underserved, ethnically diverse older adults.

**Methods:**

Seventy-one older adults were recruited through community agencies to participate in seven ethnic-specific focus groups: American Indian/Alaska Native, African American, Filipino, Chinese, Latino, Korean, and Vietnamese. Groups were conducted in the participants' primary language and ranged in size from 7–13 participants. Mean age was 71.6 years (range from 52 to 85 years; SD ± 7.39). Professional translators transcribed audiotapes into the language of the group and then translated the transcript into English. Transcripts were systematically reviewed using content analysis.

**Results:**

Suggested features of physical activity programs to enhance participation among ethnically diverse minority older adults included fostering relationships among participants; providing culture-specific exercise; offering programs at residential sites; partnering with and offering classes prior to or after social service programs; educating families about the importance of physical activity for older adults and ways they could help; offering low- or no-cost classes; and involving older adults in program development. Walking was the exercise of choice across all ethnic groups. Health served as both a motivator and a barrier to physical activity. Other factors influencing physical activity were weather, transportation, and personal safety.

**Conclusion:**

Findings from this study suggest strategies for culture-specific programming of community-based physical activity programs.

## Introduction

Because of the known health benefits of physical activity, increasing physical activity is a goal of *Healthy People 2010* (1). The health benefits of physical activity for older adults are well documented (2,3). Moderate levels of physical activity have been shown to reduce the risk of dying from heart disease (4), reduce the symptoms of depression and anxiety (5), and assist in managing chronic diseases such as diabetes and hypertension (2,6). In spite of this evidence, many older adults remain physically inactive (2).

Ethnic minority communities in the United States experience a high prevalence of chronic diseases that may be prevented or ameliorated by physical activity. African Americans, Latinos, American Indians, and Filipinos have a higher incidence than whites of diabetes, hypertension, stroke and overall mortality (7-9). Yet adults from ethnic minority groups engage in leisure-time physical activities less frequently than do adults in the rest of the population (2,10-12). Furthermore, Crespo et al found that physical activity was positively associated with acculturation (10). Often, older adults in immigrant communities are less acculturated and are therefore more likely to remain sedentary.

Despite the known benefits of physical activity and the health needs of ethnic minorities, information is limited on factors that encourage older, ethnic minority adults to be physically active (13). Little is known about how these communities perceive physical activity and the factors that encourage or discourage individuals from being physically active.

To better understand the needs and desires for physical activity programs among older, ethnic minority adults, we conducted focus groups with older adults from seven cultural groups, including five groups of older immigrants. The purposes of the study were to 1) identify barriers and facilitators to engaging in physical activity and 2) broaden our understanding of culturally appropriate physical activity and exercise programs.

## Methods

Focus groups were conducted with older adults to explore the motivations and barriers of physical activity within each of seven cultural/linguistic groups: American Indian and Native Alaskan, African American, Vietnamese, Cantonese-speaking Chinese immigrants from Vietnam, Korean, Tagalog-speaking immigrants from the Philippines, and Spanish-speaking immigrants primarily from Mexico, and also from El Salvador, Columbia, Nicaragua, Peru, and Equador. The focus groups were conducted in the primary languages of the participants. Participants were recruited from local community agencies and represented large minority communities in the Seattle area, as well as groups that have been typically underserved by existing programs promoting physical activity. Recruitment took place in October 2002, and focus groups were conducted during November 2002 through February 2003.

Four community agencies partnered with the university-based research team. These four community partners were social and health service providers that meet the needs of the following ethnic groups: Asian Americans and Pacific Islanders, African Americans, American Indians, and Latinos. Representatives from each of the agencies met with the research team to strategize the implementation of the focus groups and to develop a discussion/interview guide. Agency representatives identified facilitators and note takers for each focus group from staff members who were culturally and linguistically competent.

### Participants

Facilitators and note takers recruited participants from clients at their agency using recruitment guidelines to ensure a range of ages, varying levels of physical activity, ability to speak the language of the group, and cognitive ability for meaningful participation.

Seventy-one adults (59% women) participated in one of seven focus groups (Table 1 and Table 2). Mean age was 71.6 years (SD ± 7.39). Immigrant participants had spent an average of 15 years in the United States (SD ± 8.59). Fifty-four (76%) participants reported being able to walk 0.5 miles without help (14). Fifty-seven (80%) participants reported being able to walk up and down a flight of stairs without help (15). When asked about current exercise patterns (using the stage of exercise adoption model) (16), half reported exercising regularly and another third reported exercising some, but not regularly.

### Instrument

Facilitators used the interview guide (Appendix) that was collaboratively developed by the partnering agency representatives and the research team. For the non-English–speaking groups, facilitators translated the interview guide and the note takers checked the translation. During the group sessions, facilitators explained that for the purposes of discussion, physical activity would include everything from formal exercise to gardening and household tasks.

### Procedures

Facilitators and note takers attended a one-day training session focusing on learning about the research study and the role of physical activity for older adults, in addition to developing skills to facilitate a focus group. Training was held during the day at a convenient central location. Upon approval from the University of Washington Institutional Review Board, study personnel from the partnering agencies invited older adults from their client base to attend the focus group. Focus groups were audiotaped. Although transportation was not provided, the focus groups were held in locations frequently used by the participants, such as meeting rooms of community agencies or senior center meal sites. Notes from note takers provided backup in case of recording equipment failure. Each focus group meeting lasted 60 to 90 minutes. Participants were each given a $25 honorarium.

### Analysis

Professional translators transcribed the audiotapes into the language of the group and then translated the transcript into English. QSR NVivo qualitative analysis software (QSR International Pty Ltd, Melbourne, Australia) was used to organize the data. Members of the research team representing several disciplines — including cultural anthropology, nursing, social work, and public administration — systematically reviewed the translated transcripts, coding them for emerging themes. The team members had expertise in aging, exercise, and community-based participatory research.

Initially, all research team members reviewed and coded one of the transcripts. The team then met to review and discuss their coding. This discussion across disciplines provided a framework to review and code the remaining transcripts. Subsequently, each team member chose one of the remaining transcripts to code, with the coding then reviewed and discussed by all of the research team members. The research team invited the facilitator and note taker from each of the groups to participate in the discussion of the transcript coding. A consensus was reached on coding each transcript.

Major themes emerged after reading and discussing the transcripts, coding reports, and summaries. A draft report of the results was sent to facilitators, note takers, and other representatives from the partner agencies. The research team convened a meeting of community partners to elicit feedback on the draft results and to enrich the interpretation of findings, including ideas for potential programming.

## Results

### Common themes

#### Physical activity as health promotion

A common thread across groups was that exercise is one component of health promotion along with proper nutrition, caring for ones' emotional health, keeping the mind active, and socializing. Walking, both as exercise and as a mode of transportation, was the physical activity of choice across all groups. Participants frequently mentioned both health and social benefits as motivating factors for being physically active, especially as these factors related to managing chronic conditions such as diabetes, arthritis, hypertension, and pain. When physically active, participants felt stronger, healthier, and more energetic. One participant from the Tagalog group said, "Exercising and walking gives you energy. That's how you strengthen your body. Your weakness disappears when you walk a lot."

#### Complex role of chronic conditions

Paradoxically, both the key motivator and primary barrier for physical activity were related to health and chronic conditions. Participants cited examples of how physical activity helped them to manage chronic conditions. Chronic conditions, however, also hindered many from being physically active. Some reported not being physically active when sick or injured. Additionally, participants were aware that psychological health impacted desire and motivation to be physically active.

#### Family as encouragement

Children and other family members helped participants to be physically active by purchasing exercise equipment to use at home, transporting participants to programs, or providing encouragement. One African American participant said, "My son bought me a walk odometer, so I can tell how far I walk." Participants also kept active to remain healthy so they would not burden family members. One Korean participant commented, "My being sick makes my children suffer."

#### Environmental barriers

Environmental factors that hindered participants from being physically active or required modification of physical activity included weather; neighborhood safety; fear of crime; program costs; and inadequate availability, frequency, and reliability of affordable transportation. All groups made some reference to barriers, but some offered solutions.

### Ethnic-specific themes

#### American Indian/Alaska Native

A history of oppression and the resulting poverty and low self-esteem were common threads throughout the American Indian/Alaska Native (AI/AN) group. Low self-esteem was associated with lower motivation for self-care, including physical activity. In addition to walking, the AI/AN participants frequently mentioned providing care to other seniors as a common activity. The group expressed feelings of being disconnected and isolated from other AI/ANs; being out of place; not fitting in; and being uncomfortable around others who are non-Indian: "When you see people using fitness facilities, you see people who don't look like me. It would help if there were a group of elders who look like me." One participant wanted to serve as a role model and give his "children and grandchildren someone to emulate." Participants in the AI/AN group reported that living with chronic conditions, such as diabetes, had raised their awareness of the need to be active and lead a healthier lifestyle.

Participants in the AI/AN group were enthusiastic about the idea of getting together regularly to discuss their health concerns and to encourage each other to be active. They expressed a strong desire to be around people of similar background and identity. The cultural and community connection was seen as very important and as a motivator for participation.

#### African American

The strongest theme from this group was that of friends encouraging each other to be regularly active. "It's nice to have a friend, because if you don't feel like going, she might say something to encourage you. Or she might be after you so much that you say, 'Oh, yeah, I'll go.' And you feel so much better afterwards. Believe me." The social aspect of programs was seen as important: "Try to find yourself someone to do it with. If you can find two people to get together and one motivates the other." Participants favored group activities, although it was important to allow for individuality within those activities: "But I don't do all of what they do. I'm my own boss."

Participants understood the current recommendation of exercising a total of 30 minutes a day in shorter cumulative intervals: "You can walk for 30 minutes a day or go about five to 10 minutes, and then go back home, and later on do the same thing. I read this in a book." Walking had been a common activity when many participants were younger, and the activity served as a stress reliever, a time for meditation, and an opportunity to be in nature: "It might sound silly, but I walk and pray. When you are in nature, you find yourself grateful to be alive."

Several participants spoke enthusiastically about determination: "Main thing, you don't get lazy and you don't give up. You gotta have determination." In addition, they mentioned that exercise becomes habit forming and self-sustaining: "Exercise gets to be a part of you." In spite of the perception that damp weather could aggravate physical conditions, participants were adamant that rain would not prevent them from getting outside and getting exercise. Humor was expressed: "Girl, that rain won't melt you! You are not sugar or salt."

#### Cantonese-speaking Chinese

The importance of a daily activity routine was a prominent theme within this group. Many spoke of waking in the early morning and having a routine of stretching, arm swinging, tai chi, walking, or a combination of these. Several spoke of exercising in short increments several times a day. They viewed exercise as a critical part of maintaining health for older adults, even more important than taking medication. Participants viewed physical activity as helpful for digestion, blood circulation, relaxation, maintaining friendships, avoiding medication, preventing sickness and chronic pain, living longer and happier lives, and maintaining overall good health: "The most important reason for doing exercise every day is for health. It is only with health that you can have longevity." In addition, participants spoke of the emotional and social benefits of exercise: "Exercise makes people happy and stop thinking about anything meaningless." Another participant said, "Walk for a few bus stops, leave my troubles behind."

This group reflected a certain practicality in their responses. For example, when the weather was good, they engaged in activities such as yard work. When the weather was bad, participants spoke of indoor alternatives. Furthermore, dressing appropriately allowed participants to walk outside in the rain. However, snow was viewed as more problematic because of the fear of falling and subsequent injury. Furthermore, social obligations could interfere with an exercise routine when an unexpected visit by a friend would interrupt an exercise session.

#### Korean

Similar to the Chinese group, Koreans spoke of the importance of a daily physical activity routine. The health benefits of physical activity served as a motivation to be active: relieving joint pain, aiding digestion, and feeling more relaxed and happy: "We must walk after each meal. It helps digestion and keeps our joints flexible." Some thought being physically active would make pain worse, but they found that, in fact, it offered relief. Some felt that exercise cured their diseases. Participants identified feeling tired and dizzy as reasons for limiting physical activity. Health care providers told participants they should not walk because of their age or health condition. Similar to the AI/AN group, Koreans expressed feelings of isolation from other Koreans, including feelings of isolation even when surrounded by other Asian American groups. "In our apartment there are only Chinese women. There is not a single Korean."

#### Tagalog-speaking Filipinos

The importance of community, laughter, and socializing emerged from this group: "All of us are happy because there's laughter, storytelling, someone wins, someone loses. When we go home, we sleep soundly because there was laughter, and we played bingo." Physical activity is part of a bigger social picture. Exercise was perceived as important to counteracting the high-fat diet in the United States, which participants believed has led to increased high blood pressure among immigrants. Similar to the Cantonese-speaking group, members of the Filipino group expressed the belief that exercise aids digestion and blood circulation: "The blood is able to circulate in the person's body so the person becomes active on that day." As with other groups, a major focus was walking. Participants also mentioned stretching, tai chi, and household chores: "Before eating, I do tai chi because it's slow. That's ideal for seniors, no sudden movements."

Many Filipino participants were involved in either paid or volunteer work, such as serving as senior companions, providing childcare, and doing janitorial work. In addition to being able to send money back to the Philippines and helping others, they described how their work kept them physically active and provided enjoyment. They cited family and work obligations, however, as factors that interfered with maintaining a physical activity routine.

The Filipino group agreed that physical activity made them strong, healthy, and energetic. As with the Koreans, the Filipinos were motivated to exercise because it stimulated their appetites. They also felt younger when they were physically active*: *"Dancing makes you feel young and you never give up hope."

As with other groups, the Filipinos identified feeling physically bad or having an illness as barriers to physical activity. Barriers mentioned also included vision impairment and fears of tripping or falling. Although other groups verbalized safety concerns, the Filipinos expressed dramatic fears: rape, robbery, kidnapping, or being the target of a terrorist. Some felt they did not live in safe areas or were fearful of getting lost.

Feeling out of place when physical activities predominantly involve younger people, these older adults reported that socializing with other Filipinos of similar age was important. They spoke of building a Filipino center, with the values of unity, equality (no distinction between rich and poor), and cooperation. This group also spoke of providing peer instruction for others in the community: "We could share the exercise with those who still don't know it. We would go to those who do not leave their apartments."

#### Spanish-speaking Latinos

Because faith was an integral part of the daily activities of Spanish-speaking older adults, they brought elements of their faith to many aspects of the discussion about physical activity. One participant said, "When I wake up, the first thing that I do is to pray to God. The second, I exercise." The Latino participants emphasized music, singing, and dance as ways to remain physically active. Socializing, avoiding depression, and being outdoors were motivators for physical activity. "Activity is important because I don't get depressed...the problems in life can get you depressed."

The primary barrier to physical activity was not having a friend with whom to engage in physical activities. Similar to the Korean group, this group also mentioned dizzy spells and lack of energy as interfering with being active. This group and the Filipino group were the only two to identify visual and hearing impairments as barriers to physical activity.

#### Vietnamese

Vietnamese older adults strongly emphasized a consistent routine of daily exercise. Similar to the Cantonese and Filipino groups, participants viewed physical activity and massage as important to blood circulation. Vietnamese participants spoke of being in good health and active in spite of their age. By remaining physically active, participants said, they could avoid medication use. One participant commented, "Whether or not you are old or young, if your muscles are not stiff, you will have good health. If you are lazy and your muscles are stiff, you will become weak and you aren't able to do anything. It is a matter of daily activity and it must be consistent." One man said, "Even though I am 65 years old, I still work as a newspaper deliverer. Every morning, I wake up very early to get some physical activity. In addition to physical activities, mental activities are required. I need to remember where to deliver the newspaper, where to stop. I see this job helps me to earn money and have a comprehensive physical activity. It is really an opportunity for me." Physical activity was cited as helping with longevity: "My doctor said that my blood pressure would be very high if I don't exercise. He also said that I will die earlier if I don't do exercise. My motivation is being afraid of early death." Additionally, personal determination and willpower were named as necessities for remaining active: "Even though my doctor recommended doing so, I still need to be determined. But the limitation is your motivation. If you are lazy and unmotivated, you cannot do it."

Participants in the Vietnamese group identified geographic isolation as a barrier to physical activity. Participants lived too far from friends or too far from a park or other acceptable places to walk. Similar to the Chinese group, this group had practical responses to weather-related barriers. If it rains, they expressed that they can use indoor exercise equipment, walk in an indoor shopping mall, or do housework. Participants perceived cold weather as more problematic than rain when exercising, citing that it is difficult to breathe in cold weather.

### Components of an ideal physical activity program

Ideal programming for physical activity and exercise for older adults from multicultural groups would be "a paradise for seniors" as stated by one Chinese participant (Table 3). Budget realities may limit an organization or program's ability to provide culture-specific services and activities for each cultural group served; therefore, organizations and program developers may want to explore ways to cater to more than one cultural group.

## Discussion

Results of this study reveal that although there are ethnic-specific variations in factors influencing physical activity, there are more common themes than variations. Within an ecological model (17), patterns of health and well-being and physical activity are affected by a dynamic interaction among biological (e.g., health, disease, chronological age), psychological (e.g., enjoyment, self-efficacy, motivation, personal safety, fear of falling), social (e.g., social support, companionship, family involvement), and environmental (e.g., weather) factors. In addition, behaviors and habits (e.g., exercise history, readiness for activity) play a role in the ecological model. The interaction of these factors unfolds over the life of the individual, family, and community. Participants talked about being active as youngsters and continuing to be active as older adults. An individual's internal programming for a physically active lifestyle starts at an early age. Nies et al similarly noted the important role of internal and external contextual influences on developing and maintaining physical activity (18).

Knowledge of modifiable factors such as motivation and attitudes may help to develop interventions with the ultimate goal of changing behavior and influencing outcomes. Similar to our findings, Eyler et al found that lack of motivation was a common barrier to increasing physical activity (19). Self-motivation may reflect the presence of self-regulatory skills such as goal setting, self-monitoring of improvement, and self-reinforcement, all of which have been found to be critical for maintaining physical activity (20).

It is interesting to note that whereas lack of health contributes to sedentary lifestyles, lack of health also serves as a motivator to become more physically active. Changes in health status, therefore, may serve as cues to adopt a healthier lifestyle. In contrast to other studies that explore barriers and enhancers to physical activity (in which a determinant must be one or the other), this study found that certain factors, such as one's physical health, could serve as both barriers to and enhancers of physical activity. Similar to other studies (21,22), our participants universally identified both physical and mental health benefits of physical activity and exercise.

Our findings that frequent barriers to physical activity for older adults include personal factors (e.g., health concerns, lack of personal safety, lack of ethnic-specific exercise facilities) and environmental factors (e.g., inclement weather, transportation, costs) are similar to findings from other studies (19,23). The affinity for solving the problems associated with these barriers was encouraging. For example, although weather might have hindered physical activity, many older adults executed another plan during inclement weather (e.g., instead of walking outdoors, they walked in malls; instead of gardening in their yards, they danced indoors).

Several factors limit the generalizability. First, the standard wisdom in focus group research is to conduct at least three focus groups for each group represented to saturate the data (24), but because of budget limitations, each cultural group had only one focus group. Although common themes emerged across the spectrum of cultures, additional studies of each cultural group would provide a deeper understanding of the motivators and barriers to physical activity in these communities. Second, because of language and cultural considerations, the same facilitator was not used for all seven groups. Although training was provided, the different styles and experience levels of the facilitators may have elicited different types of information in each group. Simply because a theme was not discussed by the participants does not mean that it did not hold significance for that group. Third, the sample was drawn using a convenience sampling method and from a pool of older adults who were already using services provided by community agencies. Thus, the participants do not necessarily represent all older adults in their ethnic groups.

Despite these limitations, this study has important findings. Although there is interest in helping older adults adopt an active lifestyle, much of the published research continues to focus primarily on mainstream culture. This study used focus groups in the participants' first languages, allowing for an easier exchange of ideas. Rather than using one ethnic group, this study was able to compare results across seven ethnic groups. Also, participants in this study contributed important information about barriers and motivators in addition to specific recommendations for tailoring physical activity programming to multicultural audiences. Although generalizations cannot be made over the broader population, these groups have generated implications for practice that warrant further exploration. Knowledge of perceptions among older adults about motivators, barriers, and personally meaningful outcomes to physical activity is an essential first step to developing programs tailored to the values of each cultural group.

Future research could address several questions. To what extent does gender make a difference in being physically active as an older adult? This is particularly intriguing because men and women play different societal and cultural roles within most ethnic groups (25). What are the differences in motivators and barriers between more able-bodied older adults and those who are physically impaired? What are the differences between sedentary and non-sedentary older adults? Which aspects of peer-supported programs are critical for success?

The importance of addressing the lack of physical activity among older adults in the United States is heightened by the increasing numbers of older adults, the pervasiveness of sedentary lifestyles in this age group, and the frequent barriers to activity. Listening to voices from multiple cultures, addressing barriers, and tailoring activity programs to meet unique needs is a promising approach to improving the health and well-being of the increasingly large numbers of underserved, ethnically diverse communities of older adults.

## Figures and Tables

**Table 1:** Characteristics of Participants in a Study on Physical Activity and Exercise Among Underserved Ethnically Diverse Older Adults (n = 71), Seattle, Wash, 2002-2003^a^

		**AI/AN (n=8)**	**Af Am (n=9)**	**Can (n=9)**	**Kor (n=11)**	**Span (n=13)**	**Tag (n=11)**	**Viet (n=10)**	**Total (n=71)**
**Female **		4	6	6	6	9	7	4	42
**Male**		4	3	3	5	4	4	6	29
**Age (years)**	**Mean**	66.6	71.3	69.4	77.1	71.7	74.5	68.4	71.6
	**Range**	58-75	52-83	64-74	66-85	65-84	61-85	58-78	52-85
	**Median**	65.5	77	69	77	71	75	68	72
	**SD**	6.55	10.58	3.71	6.04	5.72	8.03	6.17	7.39
**Length of time in United States (years)**	**Mean**	NA	NA	14.6	18.3	13.1	14.3	13.8	14.8
	**Range**	NA	NA	6-22	12-30	0-46	2-27	8-27	0-46
	**Median**	NA	NA	14	18	8.5	15	11	15
	**SD**	NA	NA	4.69	5.52	13.89	8.05	6.68	8.59
**Age at immigration (years)**	**Mean**	NA	NA	54.8	58.8	58.7	60.3	54.6	57.6
	**Range**	NA	NA	51-67	47-72	34-72	40-77	35-66	34-77
	**Median**	NA	NA	54	61	59	59	57	57

a NA indicates not applicable. AI/AN indicates American Indian/Alaska Native, Af Am indicates African American, Can indicates Cantonese, Kor indicates Korean, Span indicates Spanish-speaking, Tag indicates Tagalog-speaking Filipinos, Viet indicates Vietnamese.

**Table 2:** Physical Abilities of Participants in a Study on Physical Activity and Exercise Among Underserved Ethnically Diverse Older Adults (n = 71), Seattle, Wash, 2002-2003^a^

	**AI/AN (n=8) No. (%)**	**Af Am (n=9) No. (%)**	**Can (n=9) No. (%)**	**Kor (n=11) No. (%)**	**Span (n=13) No. (%)**	**Tag (n=11) No. (%)**	**Viet (n=10) No. (%)**	**Total (n=71) No. (%)**
**Able to walk half mile without help^b^**	7 (87.5)	6 (66.7)	6 (66.7)	10 (90.9)	7 (53.8)	8 (72.7)	10 (100)	54 (76.1)
**Able to walk up and down a flight of stairs without help^c^**	8 (100)	7 (77.8)	5 (55.6)	10 (90.9)	9 (69.2)	9 (81.8)	9 (90.0)	57 (80.3)

**Stages of exercise adoption^d^**								

**Regular exerciser for more than 6 months**	4 (50)	1 (11.1)	9 (100)	3 (27.3)	2 (15.4)	5 (45.5)	7 (70)	31 (43.7)
**Regular exerciser for less than 6 months**	1 (12.5)	1 (11.1)	0 (0)	0 (0)	1 (7.7)	0 (0)	1 (10)	4 (5.6)
**Exercise some, but not regularly**	1 (12.5)	3 (33.3)	0 (0)	4 (36.4)	9 (69.2)	4 (36.4)	2 (20)	23 (32.4)
**Not an exerciser, but planning to start within 6 months **	1 (12.5)	4 (44.4)	0 (0)	4 (36.4)	1 (7.7)	0 (0)	0 (0)	10 (14.1)
**No intention to start exercising within 6 months**	1 (12.5)	0 (0)	0 (0)	0 (0)	0 (0)	2 (18.2)	0 (0)	3 (4.2)

a AI/AN indicates American Indian/Alaska Native, Af Am indicates African American, Can indicates Cantonese, Kor indicates Korean, Span indicates Spanish-speaking, Tag indicates Tagalog-speaking Filipinos, Viet indicates Vietnamese.

b Strawbridge et al, 1992 (14).

c Guralnik and Simonsick, 1993 (15).

d Marcus et al, 1992 (16).

**Table 3:** Features of Physical Activity Programming to Reach Ethnically Diverse Older Adults, Seattle, Wash, 2002-2003

**Theme**	**Specific Suggestion**
**Foster relationships among physical activity participants**	Peer support or “buddy system” Option of group classes or individual instruction Share information about exercise benefits, location, and program availability Establish informal phone tree networks
**Bring programs to where people live**	Offer exercise classes at senior housing facilities Provide transportation to community programs from senior housing facilities Start a walking club at senior housing facilities
**Partner with other programs**	One-stop shopping: combine physical activity programs with meal programs, health education classes, language classes, social events, spiritual activities Provide transportation
**Provide a variety of options**	Target programs to different levels of physical abilities Provide options for both groups as well as individuals
**Offer culture-specific programs**	Target classes and programs toward older adults from similar cultural and linguistic backgrounds Recruit an instructor who speaks the language, communicates well, and holds similar values as the older adults Weave components of the culture, such as music or traditional dance, into the program

## Appendix

**
Interview Guide Questions for a Study of Older Adult Perspectives on Physical Activity and Exercise, Seattle, Wash, 2002-2003**

1. What does being physically active mean to you?

2. Describe what you do on a regular basis that involves physical activity.

**Probe:** Think of things that you do like household chores, home repairs, yard work, walking to the store or the post office, and how you spend your leisure time.

**Facilitator:** "For purposes of this study, when we use the terms 'physical activity' or 'exercise,' we mean those activities such as gardening, yard work, vigorous cleaning, walking (including walking as a way to get to places where you need to go), swimming, dancing, yoga, and tai chi. As you are thinking about the following questions, reflect on your own life and what has encouraged or discouraged you from being physically active."

3. What motivates you to do the kinds of physical activity you currently do?

**Probes:**

- What benefits do you get from being active physically?
- Why is it important to you to keep physically active?

**Probe motivations:**

- Health
- Appearance
- Emotional well-being
- Being able to play with my grandchildren
- Have done it in the past
- Being with others
- Getting out and seeing people
- Walking to get somewhere
- Enjoy going to a park in the neighborhood

4. What has kept you from being as physically active as you would like to be? Describe those circumstances.

**Probes:**

- Safety concerns in the neighborhood
- Lighting
- No sidewalks
- Weather (cold/heat/rain)
- Traffic
- Neighborhood too hilly
- Physically unable
- Fear of injuries or falls
- Lack of interest or motivation
- Lack of money
- Lack of transportation
- Language barriers
- No one to do it with
- Places you need to go to are too far away to walk

5. If you could imagine the ideal program that would encourage you to be physically active, what would it look like?

**Probes:**

- A program that you would do on your own or in a group setting?
- Outside or inside a building or both?
- Number of days a week?
- Duration of the class?
- Characteristics of the instructor?
- Cost?
- Proximity to home (how far would you be willing to travel)?
